# Do Prenatally-Conditioned Flavor Preferences Affect Consumption of Creep Feed by Piglets?

**DOI:** 10.3390/ani9110944

**Published:** 2019-11-10

**Authors:** Jaime Figueroa, Ignacio Marchant, Pía Morales, Laura C. Salazar

**Affiliations:** 1 Departamento de Ciencias Animales, Facultad de Agronomía e Ingeniería Forestal, Pontificia Universidad Católica de Chile, Santiago 7820436, Chile; laurasalazarhofmann@gmail.com; 2 Departamento de Fomento de la Producción Animal, Facultad de Ciencias Veterinarias y Pecuarias, Universidad de Chile, Santa Rosa 11735, La Pintana, Santiago 8820000, Chile; ignacio.marchantv@gmail.com; 3 Floramatic S.A, Av. Marathon 1989, Ñuñoa, Santiago 7750000, Chile; pia.morales@floramatic.com

**Keywords:** Creep feeding, flavors, learning, piglet performance, prenatal exposure

## Abstract

**Simple Summary:**

In pig farming, weaning is abrupt, and occurs between the third and fourth week of age. Creep feed is commonly offered to reduce feed neophobia by creating a sensory link between suckling and post-weaning diets. However, low intake of creep feed is observed within and between litters. This experiment estimates the effect of prenatal flavor exposure on the performance of piglets when the same flavors are included into their creep feed. Gestational sows were fed either a flavored (garlic or aniseed; n24) or an unflavored diet (n24) from 90 to 114 days of gestation. Their litters were offered either garlic, aniseed or unflavored creep feed during the suckling period, and its intake was measured as well as the animal’s final body weight (BW) and average daily gain (ADG). No differences are found in any parameter analyzed according to prenatal flavor exposure (*p* > 0.05), observing a huge feed intake variability between litters. An unclear post-ingestive benefit of flavored creep feed intake, and the reward contrast between maternal milk and creep feed diets, may accelerate flavor learning extinction during the suckling period. Results of the present and previous experiments suggest that current flavor continuity strategies that are proposed for pig production systems need to be reconsidered.

**Abstract:**

Piglets can prefer flavors that are presented to pregnant sows, hence this study intended to estimate the effect of prenatal exposure of piglets to flavors on the intake of flavored creep feed and performance. Gestational sows were fed either a flavored (garlic or aniseed; n24) or an unflavored diet (n24) from days 90 to 114 of gestation. Their litters were offered either garlic, aniseed, or unflavored creep feed during the suckling period, and its intake was measured as well as animal’s final body weight (BW) and average daily gain (ADG). Data was analyzed taking into account the diet offered to both sows and piglets, as well as the interaction between these variables. As previous experiments have shown, flavor learning did not affect a piglet’s performance during the suckling period, finding no differences for creep feed intake, ADG, or BW according to diets (*p* > 0.05). Repeated exposure of piglets to previously learned flavors without clear post-ingestive benefits could nullify the strategies proposed. Moreover, milk and creep feed diets show a great reward contrast that may accelerate learning extinction. Results of the present and previous experiments suggest that current flavor continuity strategies that are proposed for pig production systems need to be reconsidered.

## 1. Introduction

Piglets are weaned earlier and more abruptly in intensive production systems than wild or feral swine—commonly as soon as 21–28 days of age [1]; thus it is possible that such an early weaning may interfere with the normal learning process of piglets about the foods that are safe, which normally occurs at this stage of their lives. This results in reluctant piglets, who approach unfamiliar feeds fearing they might be poisonous, and ultimately leads to poorer scores in production parameters [2,3]. A frequent strategy to mitigate food neophobia in weaned piglets is to offer them creep feed while they are still suckling, as it not only helps in preparing their digestive tract for digesting non-dairy feeds [4,5] but also familiarizes them with the novelty of solid feeds [6]. Creep feeding has also shown additional benefits in production parameters, such as greater feed intake and weight gains after weaning among those piglets who ate it [7]. However, creep feed intake among suckling piglets varies widely between and within litters, as well as among studies [8].

The pig industry is working on increasing the consumption of creep feeds by using different strategies, as advancing the start of its administration [9], changing the presentation method to stimulate feed exploration [10,11] and incorporating different additives that may improve their palatability [12]. Nevertheless, including new flavors could trigger a counterproductive effect, decreasing food intake by animals due to neophobia [3]. A possible way to either increase the proportion of piglets that consume creep feed, or to increase the amount consumed by each piglet, is via prenatal exposure to some of the flavors that are actually present in creep feeds. Arias and Chotro [13] described that when rat mothers ingest foods which include the desired flavors during late gestation, the amniotic fluid would act as an unconditioned stimulus (US) that either generates a preference or an increased acceptability for the associated flavor cues (i.e. the conditioned stimulus, CS). In this regard, researchers previously observed that when piglets were exposed in the prenatal stage to some flavors, they did prefer those flavors latter on, during the suckling period [3], and these animals also presented a higher feed intake and body weight when those flavors were included in their post-weaning diets [14]. Interestingly, such a scenario could also lead to positive effects on animal welfare [15]. However, no clear information is reported regarding the effect that any prenatal learning of flavor preferences might have on feed intake and the performance parameters of suckling piglets [3,16]. In light of the information discussed so far, this study intended to estimate whether prenatal exposure to flavors shows any effect on both creep feed intake and the growth rate of suckling piglets.

## 2. Materials and Methods

A commercial intensive pig farm located in the Libertador Bernardo O’Higgins region in Chile was selected to perform this study. All experimental procedures were approved by the Bioethics Committee of the “Facultad de Ciencias Veterinarias y Pecuarias”, Universidad de Chile (Certificate No 252014). 

### 2.1. Animals and Housing

A total of 48 sows and their litters (PIC^®^ genetics, Hendersonville, Tennessee, USA), were used in this study between day 90 of pregnancy (late gestation) and day 21 after farrowing, to measure the effect of prenatal exposure to flavors on piglets’ performance, such as creep feed intake, body weight (BW) and average daily gain (ADG). Sows were housed under controlled temperature conditions, first in single, conventional gestation crates (23–25 °C), and then in single, conventional farrowing crates (28–30 °C) with automatic ventilation. Their piglets had access to a heated area, and stayed with sows and littermates in farrowing crates throughout the whole study. Animals were cross-fostered within treatments before day 3 after farrowing to equilibrate litters in the number of piglets. No environmental enrichment was delivered to sows or piglets during the gestation and nursing periods, respectively, following the guidelines of that farm. Pregnant sows were subjected to feed restrictions until day 112 of gestation by offering them only 1.8–3.5 kg/day of a commercial diet suitable for their body condition. 

On day 112, sows were placed in farrowing crates and stayed in there up to weaning time. Both sows and piglets were individually identified using plastic ear tags. Starting from an age of 10 days and up to day 20, piglets were provided with ad libitum access to experimental creep feed diets by using pan feeders. No additional oral treatment that could possibly change the perception of flavors was given. During the lactation period, following a farm’s usual feeding routine, sows were fed twice a day with a commercial, unflavored diet, considering the maximum consumption expected according to litter size and the sow’s body condition. Sows and piglets were also allowed ad libitum access to fresh water, which was delivered by nipple drinkers throughout the whole experimental period. Once the experiment ended, all pigs continued the normal production cycle of the farm. 

### 2.2. Procedure

On day 90 of gestation, sows were assigned to one of two diets (unflavored or flavored) that were offered to them for the last three weeks of gestation. The animals were selected ensuring there were no differences between groups related to their body weight (BW; 191 vs. 190 kg; *p* = 0.914; SEM = 6.88), P2 back-fat thickness (12.8 vs. 12.7 mm; *p* = 0.849, SEM = 0.3), parity number (3.13 vs. 3.14; *p* = 0.946, SEM = 0.19) and body-condition score (2.88 vs. 2.93; *p* = 0.523, SEM = 0.06). Both the unflavored and the flavored diets were based upon the same commercial formulation. The unflavored diet did not include any artificial flavor (n = 24), whereas the flavored diet was flavored either with artificial garlic (n = 12) or aniseed (n = 12) artificial flavors, which were selected according to their ability to cross the placental barrier [3,16,17], and included at a dose of 0.75 g/kg supported by company recommendations (Floramatic^®^, Metropolitan Region, Santiago, Chile). The feed restriction of sows during gestation assured the intake of all the feed and flavors delivered. 

**Figure 1:**
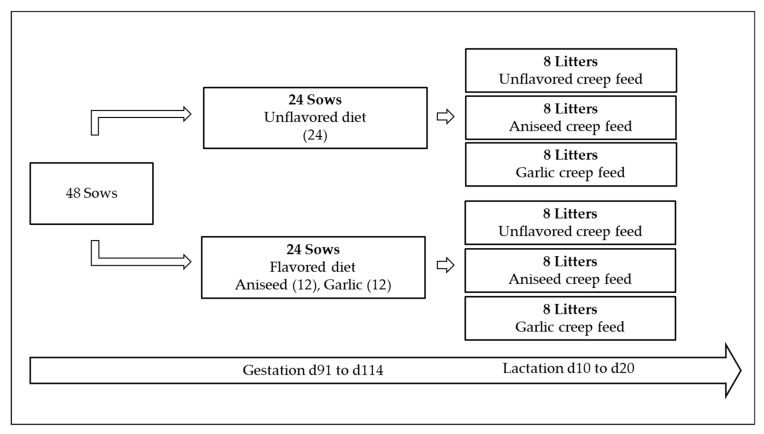
Experimental design considering flavors included in late-gestation maternal diet (days 90 to 114) and in creep feed diets for suckling piglets (days 10 to 20).

The effect of prenatal exposure to flavors in a piglet’s performance was assessed by measuring the creep feed intake, body weight, and calculating ADG after offering either unflavored or flavored creep feed diets from days 10 to 20 after farrowing. Experimental creep feed diets included the same concentration of flavors previously added to the sows’ gestational diets (0.75 g/kg). According to the diet that was fed to the sows before farrowing, their litters were assigned to one of three groups, which were fed either unflavored, garlic-flavored, or aniseed-flavored creep feed diets (see Figure 1). Then, creep feed intake was measured from days 14 to 18 of each litter to homogenize the intake of piglets with different farrowing days. This intake was calculated daily as the difference between initial and final weight measurements of the feeder of each litter. Additionally, all piglets were weighed at days 4 (initial BW) and 20 (final BW) after farrowing to calculate the ADG parameter.

### 2.3. Statistical Analysis

Daily creep feed intake (days 14 to 18), final BW (day 20) and ADG were subjected to an Analysis of Variance (ANOVA) using the generalized linear model (GLM) procedure of the SAS^®^ statistical package (SAS Inst. Inc., Cary, NC, USA). The variables considered were: Type of feed offered to pregnant sows (i.e., garlic-flavored, aniseed-flavored, or unflavored), type of creep feed offered to suckling piglets (i.e., garlic-flavored, aniseed-flavored, or unflavored) and the interaction between these variables. 

Additionally, prenatal associative learning in piglets was also studied by sorting data according to whether they were offered creep feed with the same flavor than their pregnant mothers (garlic/garlic, or aniseed/aniseed; Positive conditioned stimulus or CS+), a different flavor or neutral stimulus (garlic/aniseed, or aniseed/garlic; Neutral stimulus or NS), or an unflavored one (garlic/unflavored, aniseed/unflavored; Flavored-Unflavored or FU). Moreover, those piglets who were offered a flavored creep feed, but their mother was fed an unflavored diet while pregnant (Unflavored-Flavored or UF), were analyzed.

Before proceeding with ANOVA, the dataset was analyzed using UNIVARIATE and GLM procedures with the Shapiro-Wilk and O’Brien’s tests to assess its normality and homoscedasticity, respectively. No significant p-values were found for any specific factors (*p* > 0.10), hence the null hypotheses of normality and variance homogeneity were accepted. Mean values are presented as Least-Square means with simple effect comparisons between these means and a significance level of 5% (adjusted by the Tukey test). The experimental unit was the litter, and results were analyzed and expressed by piglet.

## 3. Results

### 3.1. Performance Parameters of Piglets

**Figure 2:**
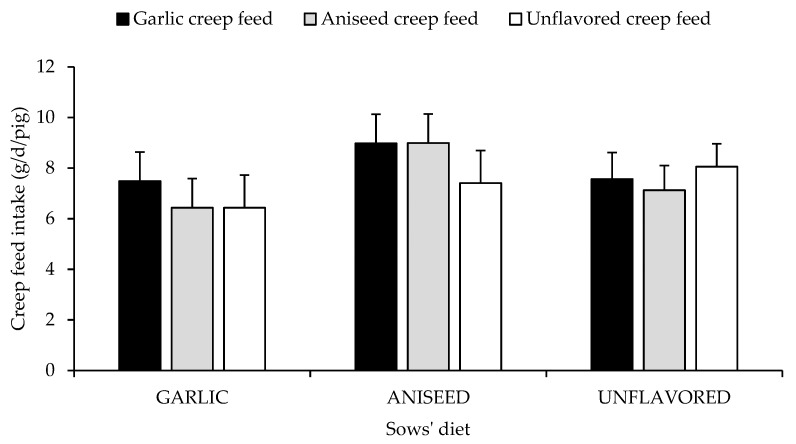
Daily intake of creep feed diet by piglets (days 14 to 18), who were offered (from days 10 to 20 of the suckling period) according to late-gestation maternal diet (GARLIC, ANISEED OR UNFLAVORED) and creep feed diet (Garlic, Aniseed or Unflavored). Results expressed as Least Square means ± SEM.

The average number of piglets per litter after the cross fostering was 11.78 ± 1.2, and their body weight was 1.78 ± 0.4 kg. Figure 2 shows creep feed intake according to flavors added into the late-gestation maternal diet and flavors added into the creep feed. No differences were found for piglets’ creep feed intake in relation with artificial flavors added during the suckling period (F_2.48_ = 0.27 and *p* = 0.766). Also, no differences were found for the interaction between the maternal diet and the flavors added to creep feed diets (F_4.48_ = 0.41 and *p* = 0.798). 

Table 1 presents piglets’ BW and ADG according to flavors added to late-gestation maternal diets and to creep feed diets. No significant differences were found in the final BW of piglets (F_2.49_ = 1.19 and *p* = 0.315), or in their ADG (F_2.49_ = 1.05 and *p* = 0.359), according to the flavors added to creep feed diets. Lastly, no interactions were observed between the maternal diet and the flavors that were added to creep feed diets over piglets’ final BW (F_4.49_ = 0.61 and *p* = 0.657) or ADG (F_4.49_ = 0.43 and *p* = 0.783).

### 3.2. Associative Learning and Sensory Continuity

**Figure 3:**
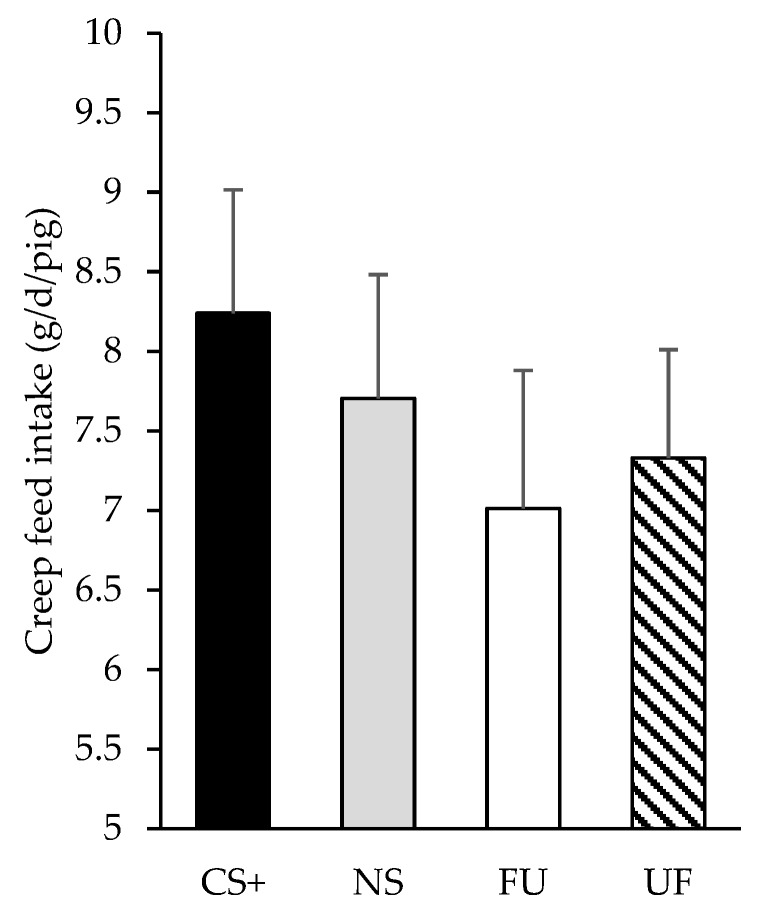
Daily intake of creep feed diet by piglets (days 14 to 18) who were offered (from days 10 to 20 of the suckling period) either the same flavor in their diet than their mothers in the late-gestation (90-114d; CS+), a different flavor (NS), an unflavored creep feed diet (FU), or a flavored creep diet, but born from a mother that was fed an unflavored maternal diet (UF). Results expressed as Least Square means ± SEM.

**Figure 4:**
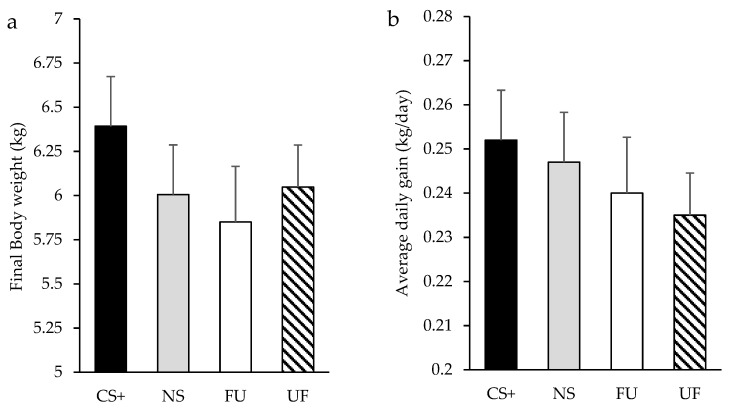
Final Body weight (**a**) and average daily gain (**b**) of piglets who were offered (from days 10 to 20 of the suckling period) either the same flavor in their diet than their mothers in the late-gestation (90-114d; CS+), a different flavor (NS), an unflavored creep feed diet (FU), or a flavored creep diet, but born from a mother that was fed an unflavored maternal diet (UF). Results expressed as Least Square means ± SEM.

Figure 3 presents the creep feed intake results according to the new data distribution in relation to prenatal associative learning (creep feed with the same flavor in their diet than their mothers in the late-gestation, CS+; a different flavor, NS; an unflavored creep feed diet, FU; or a flavored creep diet offered to piglets born from mothers that were fed an unflavored maternal diet, UF). As before, this analysis shows no differences in creep feed intake between groups, (F_3.40_ = 0.44 and *p* = 0.728). Piglet’s productive parameters in these experimental groups are presented on Figure 4. No differences were found among groups regarding ADG (F_3.41_ = 0.51 and *p* = 0.681) or final BW (F_3.41_ = 0.62 and *p* = 0.608).

## 4. Discussion

Transitioning from milk to solid feeds is a major hurdle for farm animals within commercial systems in terms of increasing, or at least maintaining, their body weight after being weaned [18]. Neophobic reactions when faced with new feeds may lead animals to poor consumption of those diets, especially if animals did not have a chance to learn new feeding behaviors from their mothers while they were still suckling [19]. 

However, evidence shows that feed preferences can be redirected—hence reducing neophobia in pigs— by the influence of flavors derived from maternal diets that are present in the amniotic fluid and milk, even when piglets are nursed for brief periods [14]. This phenomenon might be an adaptive mechanism that sows develop to help their offspring know the kind of foods that are available in their environment, even before they are able to actually experience oral and post-oral rewards [20].

Our work focuses on studying the effect of the prenatal exposure of piglets to garlic and aniseed flavors over piglets’ performance, such as creep feed intake, body weight and daily weight gain during the suckling period. The experiment measures acceptance rather than preferences as a consequence of how important is to estimate the relationship between possible prenatal learning of feed flavors and the real feed consumption that can expected from litters within the context of commercial farms. Previous works hypothesize that the associative learning that occurs during gestation (between some flavors coming from the maternal diet and the hedonic effect of amniotic fluid), would increase the consumption of foods that include those flavors in their formulation later on [13]. However, our results agree with those of previous experiments from other researchers, as we find no evidence to confirm that either the intake of creep feed or the ADG of suckling piglets improves due to any prenatal exposure to flavor cues [3,16]. 

Meanwhile, after prenatal exposure to some flavors that cross the placental barrier, a higher preference for those flavors has been reported for rats [21], dogs [20] and human infants [22]. In addition, even though some experiments that studied the prenatal learning of flavor cues in pigs actually report good results when feeding behavior and welfare were measured [3,15], most experiments do not find any significant differences regarding feed intake when production parameters are assessed in suckling piglets. Contrarily, higher feed intake, ADG and body weight are reported in weaned piglets during the nursery stage (days 0 to 33 after weaning) when these piglets were fed diets that included the same hydrolyzed protein that their mothers ate during late-gestation [14]. Moreover, Oostindjer et al. [15] described that prenatal exposure to flavor cues is associated to an overall positive effect on animal welfare after weaning (e.g. reduced manipulation and mounting of pen-mates, as well as reduced engagement in fights). However, even though Figueroa et al. [3] described a change in flavor preferences in suckling piglets, they observed no differences in regards to prenatal learning when the intake of creep feed diets was analyzed. This situation could be explained by how these variables were tested. Unlike in the creep feed consumption test (which was measured inside farrowing pens), researchers tested flavor preferences in animals by adding flavors to cotton strips in an experimental field, while avoiding both the interference of sows and milk consumption. 

The distinctive variability among litters makes quite difficult to find significant results for consumption of creep feed diets [6,8,23]. Such variability may be partially explained by size and weight, as those piglets that are smaller and lighter than their siblings eat more of their creep feed ration, so that they can manage to satisfy their energy requirements, hence compensating for the problem of lesser access to milk [6,24]. Interestingly, some evidence shows that weaning age in piglets has an effect on their actual intake of creep feed diets. For instance, researchers have reported that suckling piglets begin showing real signs of interest in solid feed after reaching 22 days old [23]. However, previous experiments showed the benefit of initiating creep feed from day 5 and 10 [9,25]. Consequently, in our experiment, creep feed was added from day 10 until day 20, as that mirrors the current practice of Chilean pig farmers, who wean their piglets after 21 days of nursing. However, European welfare regulations currently propose a weaning age of 28 days to allow that the animals increase their intake of creep feed diets, thus favoring the proper development of their digestive tract [5] and preparing pigs physiologically—as well as in terms of helping them memorize the characteristics of solid feeds—to endure the stress of weaning [15]. But this strategy seems to have some drawbacks, as it was reported by Blavi et al. [16], who found a lower intake in piglets when the formulation of creep feed diets included the same artificial flavors than the late-gestation maternal diets had, even when the nursing period was extended up to 28 days. 

This could be explained, because as nursing progresses, piglets feel less drawn to their mothers [3,26], and the attraction to amniotic-fluid-related flavor cues wanes as well, hence it becomes easier to observe prenatal flavor learning signs at the beginning of the suckling period than by the end of it.

Another possible explanation for the constant failure on using creep feed consumption to describe the prenatal learning of flavor cues, is that the conditioned preference for a given flavor could be extinguished when the conditioned stimulus (i.e. the flavor learned by prenatal exposition) stops producing a greater reward to piglets than that produced by consuming an alternate food, such as milk [27,28]. Such a scenario might happen if these flavors are included in creep feed formulations while piglets are still suckling milk. Therefore, repeated exposure of piglets to previously learned flavors, but without reaping a clear post-ingestion benefit (which would be the case if piglets do not consume enough creep feed), could end up nullifying the strategies that have been proposed to help animals learn these flavors via prenatal exposure. Moreover, the association of flavors with the hedonic effect of amniotic fluid is seemingly more susceptible to extinction over time than flavor preferences coming from a previous association with sucrose, as the unconditioned stimulus (that in addition gives a post-ingestive reward). In this way prenatal learning, as well as social learning, may require that animals experience a positive experience with those flavors when interacting with feeds, in a trial-and-error fashion, to ensure that piglets keep preferring those flavors [29,30,31]. Interestingly, Blavi et al. [16] reported an improved feed intake in weaned pigs if diets included the same flavor that their mothers were fed during the late-gestation period, but only if that flavor was not added to creep feed diets during the suckling period.

## 5. Conclusions

Pigs are able to prenatally learn flavor preferences, which is adaptive to find these flavored resources in the environment during the suckling period. However, adding flavors that were prenatally learned into creep feed diets could reduce the pig’s attraction to those flavors afterwards, because of a possible learning extinction. The present experiment and several papers confirm that prenatal exposition to flavor cues do not increase piglet’s performance when those flavors are given, reflecting that current flavor continuity strategies that have been proposed for pig production systems need to be reconsidered. 

## Figures and Tables

**Table 1:** Body weight (BW) and average daily gain (ADG) of suckling piglets according to flavors added to late-gestation maternal diets (91–114 days) and creep feed diets (from age 10 days old onwards).

	Creep Feed (Means ± SEM)		
Variables	Garlic *	Aniseed *	Unflavored
**Initial BW (day 4) (kg)**			
***Sow diet***			
Garlic	1.788 ± 0.19	1.604 ± 0.19	1.490 ± 0.21
Aniseed	1.868 ± 0.19	1.856 ± 0.19	1.853 ± 0.21
Unflavored	1.800 ± 0.16	1.720 ± 0.16	1.904 ± 0.15
**Final BW (day 20) (kg)**			
***Sow diet***			
Garlic	6.554 ± 0.34	5.912 ± 0.34	5.478 ± 0.45
Aniseed	6.098 ± 0.34	6.230 ± 0.34	6.223 ± 0.45
Unflavored	6.321 ± 0.34	5.774 ± 0.34	5.865 ± 0.32
**ADG (days 4 to 20) (kg)**			
***Sow diet***			
Garlic	0.250 ± 0.02	0.236 ± 0.02	0.220 ± 0.02
Aniseed	0.258 ± 0.02	0.254 ± 0.02	0.260 ± 0.01
Unflavored	0.249 ± 0.01	0.221 ± 0.01	0.233 ± 0.01

* 0.75 g/kg of artificial garlic or aniseed flavor, Floramatic^®^, Region Metropolitana, Santiago, Chile.
